# Radical Cystectomy with Ileal Orthotopic Neobladder after 70 Years Leads to Worse Health-Related Quality of Life

**DOI:** 10.3390/jcm13206102

**Published:** 2024-10-13

**Authors:** Nikolaos Pyrgidis, Gerald Bastian Schulz, Benedikt Ebner, Friedrich Jokisch, Lennert Eismann, Deniz Karatas, Sarah Takayama Fouladgar, Julian Hermans, Patrick Keller, Christian Stief, Yannic Volz

**Affiliations:** Department of Urology, University Hospital, LMU Munich, 81377 Munich, Germany; nikolaos.pyrgidis@med.uni-muenchen.de (N.P.); gerald.schulz@med.uni-muenchen.de (G.B.S.); benedikt.ebner@med.uni-muenchen.de (B.E.); friedrich.jokisch@med.uni-muenchen.de (F.J.); lennert.eismann@med.uni-muenchen.de (L.E.); deniz.karatas@med.uni-muenchen.de (D.K.); julian.hermans@med.uni-muenchen.de (J.H.); patrick.keller@med.uni-muenchen.de (P.K.); christian.stief@med.uni-muenchen.de (C.S.)

**Keywords:** bladder cancer, cystectomy, quality of life, neobladder

## Abstract

**Background:** Radical cystectomy (RC) with the formation of an ileal orthotopic neobladder (ONB) may adversely affect long-term health-related quality of life (HRQOL). An advanced age at the time of ONB construction could further exacerbate the decline in HRQOL. This study aims to establish an evidence-based age threshold at the time of RC with ONB, beyond which a significant deterioration in HRQOL is observed. **Methods:** We retrospectively analyzed all bladder cancer patients in our department between 2013 and 2022 that fulfilled the EORTC-QLQ-C30 questionnaire preoperatively, as well as at 3 and 12 months after RC with ONB. Patients receiving neoadjuvant or adjuvant chemotherapy or benign/palliative RC were excluded. **Results:** Overall, 120 patients (81% males) with a mean age of 66 ± 9.6 years underwent RC with ONB and fulfilled the selection criteria. The Global Health Status (GHS) of the EORTC-QLQ-C30 was 64 ± 23 preoperatively, was 64 ± 20 three months postoperatively, and was 68 ± 23 twelve months postoperatively. Overall, 80 (67%) patients presented an increase in GHS at twelve months compared to their preoperative values. The perioperative complications did not differ between patients with decreased and increased GHS. Patients with increased GHS had values of 58 ± 24 preoperatively, 67 ± 19 at 3 months, and 77 ± 16 at 12 months. Patients with decreased GHS had values of 76 ± 16 preoperatively, 57 ± 21 at 3 months, and 50 ± 25 at 12 months. Using ROC analyses with Youden’s index, we defined a threshold of 70 years, after which RC with ONB may lead to worse GHS twelve months postoperatively. Worse continence outcomes were the only perioperative and long-term parameters that predicted worse HRQOL in elderly patients. **Conclusions:** Based on HRQOL, we suggest that RC with an ileal conduit as a urinary diversion should be recommended in patients older than 70 years.

## 1. Introduction

With an annual global incidence of approximately 573,000 new cases, bladder cancer persists as the tenth most prevalent malignancy across both genders [1]. The established standard of care for muscle-invasive and very-high-risk non-muscle-invasive bladder cancer involves radical cystectomy (RC) after neoadjuvant chemotherapy [2]. Among the various options for urinary diversion, the choices commonly considered are the ileal conduit and the orthotopic ileal neobladder (ONB) [3]. As the population affected by bladder cancer ages, urologists are increasingly confronted with the decision of selecting the most appropriate urinary diversion for older patients requiring RC [4].

The determination of which urinary diversion type offers superior health-related quality of life (HRQOL) outcomes remains a contentious issue in the current scientific literature [5]. The available literature suggests that up to 80% of all patients undergoing RC with ONB are affected by incontinence or other disorders that negatively impact their HRQOL [6,7]. Age emerges as a pivotal factor influencing continence and HRQOL outcomes after RC with ONB. Particularly, the incidence of short- and long-term complications after RC increases with age [8]. Current guideline recommendations indicate that although advanced age is not considered an absolute contraindication for ONB reconstruction, it should preferably be avoided in patients older than 80 years [9]. Nevertheless, it should be highlighted that no strict contraindication for the exact age exists and that relevant studies in the field are lacking [10].

Within this scope, we aimed to determine a data-based optimal age threshold at the time of RC with OΝΒ, after which HRQOL worsens.

## 2. Materials and Methods

### 2.1. Study Design and Selection Criteria

We performed the present single-center prospective cohort study at the Department of Urology of the University Hospital, LMU Munich, Germany. The study protocol was predefined and approved by the corresponding institutional review board (reference number: 20-179). This study was conducted in accordance with the ethical principles outlined in the Declaration of Helsinki. Written informed consent was obtained from all participants, and the results were reported in compliance with the STROBE guidelines for cohort studies [11].

All study participants undergoing open RC with ONB between May 2013 and July 2022 filled out validated HRQOL questionnaires preoperatively, as well as at 3 and 12 months postoperatively. We excluded patients who underwent RC for palliative or non-oncological indications, those who received neoadjuvant or adjuvant chemotherapy within 12 months of the operation, and those who did not answer the questionnaires.

### 2.2. Data Collection and Follow-Up

The rationale of the present study has been previously described [6]. HRQOL was assessed preoperatively and at three and twelve months postoperatively using the validated German translation of the standardized European Organization for Research and Treatment of Cancer (EORTC) QLQ-C30 questionnaire [12]. The EORTC QLQ-C30 comprises thirty questions and is designed to assess patients’ cancer-related physical, psychological, and social functions, documented as functioning and symptom scores. In QLQ-C30, higher symptom scores indicate more bothersome symptoms, while higher functioning scores indicate better functioning. General HRQOL was also evaluated using the EORTC QLQ-C30 Global Health Status (GHS; questions 29 and 30 of the QLQ-C30 questionnaire). In GHS, higher values indicate a better general HRQOL. Furthermore, the International Consultation on Incontinence Questionnaire (ICIQ) was used to assess the levels of postoperative incontinence. Continence was defined in the present study as the usage of no pads per day.

### 2.3. Primary Outcome and Statistical Analysis

The primary outcome of the present study was to determine a data-based optimal age threshold at the time of RC with ONB, after which HRQOL worsens. To identify this optimal age threshold, we used Youden’s index based on the receiver operating characteristic (ROC) analysis. Youden’s index was calculated for each point of the ROC curve and its maximum value was selected as a criterion to estimate the optimal age threshold, after which GHS worsened at 12 months after surgery compared to its preoperative values. Accordingly, the sensitivity and specificity, as well as the positive and negative predictive values of the optimal age threshold after which RC with ONB should be preferably avoided, were also provided. Continuous variables were summarized as means with standard deviations (SDs), and categorical variables were summarized as frequencies with proportions. All analyses were undertaken with R statistical software (version 3.6.3, R Core Team 2020). For all outcomes, two-sided *p*-values < 0.05 were considered statistically significant.

## 3. Results

### 3.1. Baseline Characteristics

A total of 120 patients underwent RC with ONB and fulfilled our selection criteria. Their mean patient age was 66 ± 9.6 years, and 97 (81%) patients were male. The operative time was 222 ± 53 min and the intraoperative blood loss was 417 ± 367 mL. Of note, 18 (16%) patients presented positive lymph nodes (pN+) and 34 (28%) presented locally advanced bladder cancer at the time of RC.

Overall, 80 (67%) patients presented an increase in the GHS at twelve months after surgery compared to their preoperative values. Except for higher rates of diabetes in patients with an increase in GHS (*p* = 0.02), the two groups did not display any statistically significant differences in their baseline and perioperative characteristics. Notably, patients with a decrease in HRQOL twelve months postoperatively did not experience perioperative complications as often as patients with an increase in HRQOL (*p* = 0.5). Further baseline characteristics of the two groups are available in Table 1.

### 3.2. Health-Related Quality of Life over Time

The 80 patients with an increase in GHS at twelve months after surgery had GHS values of 58 ± 24 preoperatively, 67 ± 19 at three months, and 77 ± 16 at twelve months after surgery. On the contrary, patients with a decrease in GHS at twelve months after surgery had GHS values of 76 ± 16 preoperatively, 57 ± 21 at three months, and 50 ± 25 at twelve months after surgery. Accordingly, 54 (68%) patients in the increase group presented values of GHS that were higher than 70, compared to 11 (28%) patients in the decrease group (*p* < 0.001). Notably, patients with an increase in GHS presented higher continence scores at twelve months postoperatively compared to patients with a decrease in GHS. As shown in Table 2, the ICIQ score twelve months postoperatively was 7.5 ± 6 for patients with an increase in GHS and 12 ± 6 for patients with a decrease in GHS (*p* < 0.001). Similarly, the number of patients that showed any grade of incontinence was significantly higher in patients with a decreased GHS at twelve months (79% versus 95%, *p* = 0.047). Concerning further variables of the HRQOL questionnaire, there were no significant differences between the two groups in terms of functional and symptom domains twelve months postoperatively. The absolute scores of the different EORTC QLQ-C30 symptom and functioning scores preoperatively, at three, and at twelve months are summarized in Supplementary Table S1.

### 3.3. Threshold of Age at the Time of Surgery for a Reduced HRQOL

Based on the findings of our study, the optimal age threshold at the time of RC with ONB after which GHS seems to worsen at 12 months after surgery compared to the preoperative values was estimated to be 70 years. This threshold displayed a sensitivity of 48%, a specificity of 65%, a positive predictive value of 39%, and a negative predictive value of 70%.

### 3.4. Health-Related Quality of Life Outcomes Based on the Age Threshold

**Figure 1:**
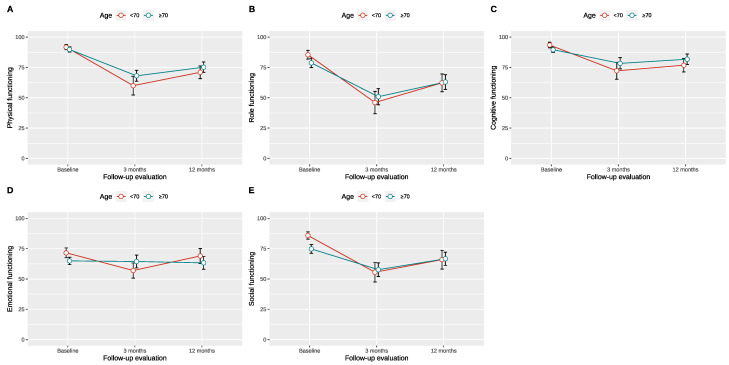
QLQ-C30 functioning scores over time based on the proposed age cutoff of 70 years for ileal orthotopic neobladder. (**A**)–Physical functioning score, (**B**)–Role functioning score, (**C**)–Cognitive functioning score, (**D**)– Emotional functioning score, (**E**)–Social functioning score.

**Figure 2:**
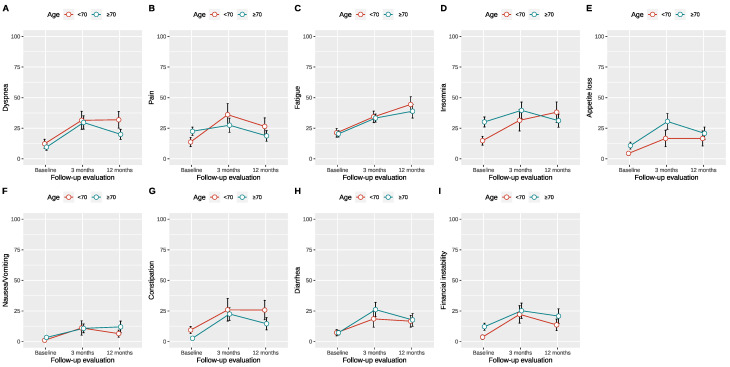
QLQ-C30 symptom scores over time based on the proposed age cutoff of 70 years for ileal orthotopic neobladder. (**A**)–Dyspnoe score, (**B**)–Pain score, (**C**)–Fatique score, (**D**)–Insomnia score, (**E**)–Appetite loss score, (**F**)–Nausea/Vomiting score, (**G**)–Constipation score, (**H**)–Diarrhea score, (**I**)–Financial instability score.

After applying the optimal age threshold of 70 years for RC with ONB, after which GHS seems to worsen at 12 months postoperatively compared to the preoperative values, significant differences were observed between the two groups regarding coronary artery disease (*p* = 0.003), ASA score (*p* = 0.018), and operative time (*p* = 0.033). Further baseline characteristics were without statistically significant differences (Table 3). When comparing the relative change from preoperative to the 12-month postoperative timepoint of the functional scores, we observed no statistically significant differences between patients above and below 70 years of age, even though patients younger than 70 years presented better absolute scores. The absolute scores of the different EORTC QLQ-C30 symptom and functioning scores preoperatively, at three, and at twelve months are presented in Supplementary Table S2. Accordingly, the change in all EORTC QLQ-C30 scores is presented graphically in Figure 1 and Figure 2, and their differences in functional scores from baseline are presented in Table 4.

## 4. Discussion

The findings of the present single-center study suggest that in patients older than 70 years, RC with ONB leads to worse GHS twelve months postoperatively. Still, two-thirds of all patients undergoing RC with ONB at our center presented an increase in GHS at the 12-month evaluation compared to their preoperative values. Importantly, no statistically significant differences in the baseline characteristics, histology findings, and in-hospital complications were identified between the patients with an increase in GHS at twelve months and the patients with a decrease in GHS, indicating that the perioperative factors do not significantly affect the HRQOL in the long term. Accordingly, from all perioperative and long-term parameters assessed, apart from age younger than 70 years, only the presence of continence seemed to be associated with better HRQOL outcomes after RC with ONB.

The available literature indicates that age plays a crucial role in predicting HRQOL and postoperative outcomes after RC with ONB [13]. Therefore, current guideline recommendations suggest that urinary diversion with ONB should be avoided after a specific age [14]. The present study is, to the best of our knowledge, the first to define, based on GHS at 12 months, an age-related cutoff, after which RC with ONB may be related to a worse HRQOL. Nevertheless, it should be noted that based on our analyses, the functional and physical scores of the EORTC QLQ-30 did not present any significant differences between patients above and below 70 years. Similarly, perioperative outcomes also did not differ between patients above and below 70 years. Therefore, it seems that these parameters may play a minor role in the deterioration of HRQOL. On the contrary, our analyses suggest that incontinence may be the driving force for a worse HRQOL in all patients.

RC with urinary diversion in patients with bladder cancer exerts a debilitating effect on HRQOL [15]. The available literature suggests that the EORTC QLQ-C30 with the different functioning and symptom scores and the GHS are useful tools to assess the patients’ overall HRQOL after RC [16]. In our study, patients with increased GHS at the 12-month evaluation timepoint did not show any significant differences in comorbidities or perioperative outcomes such as operation duration, blood loss, and tumor stage compared to patients with decreased GHS at the 12-month evaluation timepoint. Based on the previous notion, our data are in line with previous studies that have evaluated the HRQOL in patients with ONB after RC [17]. Relevant studies in the field also indicate that perioperative complications, duration of surgery, or tumor-specific variables did not seem to negatively affect the HRQOL based on the EORTC QLQ-C30 [18]. Therefore, it seems that these parameters may play a rather minor role in decreasing the HRQOL in the long term.

On the contrary, it seems that incontinence negatively affects HRQOL following RC with ONB. Previous studies on the matter suggest that incontinence after ONB is significantly associated with a reduced GHS [19]. Indeed, our findings demonstrate that continent patients, as well as patients with lower ICIQ scores, present a better HRQOL according to the EORTC QLQ-C30. In particular, only 5% of all patients with a decrease in GHS were continent after 12 months, compared to 21% of all patients with an increase in GHS. Overall, the total continence rates of the present study were rather small compared to other relevant studies that suggest continence rates in up to 58% of all patients [20]. The latter is explained by our strict definition of continence after ONB with zero pads per day. Moreover, all women underwent non-sexual-organ-preserving surgery, which might have also negatively affected the continence outcomes [21].

It should be highlighted that the findings of the present prospective cohort study were tempered by some important limitations relevant to its sample size and its single-center design. Even though we estimated a threshold after which RC with ONB may lead to a worse HRQOL based on GHS, the relatively low number of included patients did not permit us to perform regression analyses to adjust for further perioperative characteristics that might have affected outcomes. Moreover, an important amount of patients undergoing RC with ONB in our department had to be excluded from the present study. In particular, those who did not complete the questionnaires, patients who underwent RC for palliative or non-oncological indications, and those who received neoadjuvant or adjuvant chemotherapy were not included in the study to avoid rendering the interpretation of our results problematic. Importantly, we did not assess further factors that may affect HRQOL such as erectile function. Of note, the exact number of pads used daily could not be provided. Additionally, our findings may not be extrapolated to other departments that perform ONB with a different surgical approach. Finally, it should be noted that even though the threshold of 70 years seems to worsen the GHS, results regarding the EORTC QLQ-C30 functioning and symptom scores were without statistical significance. Thus, our results should only serve as a potential guideline when counseling patients aged 70 years or above for a urinary diversion with ONB.

## 5. Conclusions

The present study is the first to try to define an exact threshold based on the HRQOL after which RC with ONB should be avoided. Individuals older than 70 years seem to be at an increased risk for worsening HRQOL, potentially caused by their higher rates of incontinence. The latter should be taken into consideration when preoperatively counseling patients about the available urinary diversions after RC. It seems that in those patients, RC with an ileal conduit as a urinary diversion should be recommended.

## Figures and Tables

**Table 1:** Baseline characteristics of patients with an increase in GHS versus a decrease in GHS at twelve months after surgery. Values presented as mean ± standard deviation or *n* (%). The *t*-test was performed for comparisons between continuous variables and the chi-squared test between categorical variables. The bold cells indicate statistically significant *p*-values. ASA: American Society of Anesthesiology, BMI: body mass index, GHS: Global Health Score.

Characteristic	Overall, *n* = 120	Increase in GHS, *n* = 80	Decrease in GHS, *n* = 40	*p*-Value
Male	97 (81%)	65 (81%)	32 (80%)	>0.99
Age (years)	66 ± 9.6	66 ± 9.7	66 ± 9.6	>0.99
BMI (kg/m^2^)	27 ± 4.3	27 ± 4.3	27 ± 4.3	0.63
Smokers	75 (65%)	47 (61%)	28 (72%)	0.35
Alcohol consumption	48 (41%)	30 (38%)	18 (46%)	0.55
Hypertension	54 (45%)	37 (46%)	17 (43%)	0.85
Diabetes	33 (28%)	16 (20%)	17 (43%)	**0.017**
Coronary heart disease	18 (15%)	12 (15%)	6 (15%)	>0.99
ASA score				0.23
1	4 (3.4%)	2 (2.5%)	2 (5.3%)	
2	40 (34%)	31 (39%)	9 (24%)	
3	74 (63%)	47 (59%)	27 (71%)	
Operative time (minutes)	222 ± 53	222 ± 52	222 ± 55	>0.99
Blood loss (ml)	417 ± 367	401 ± 370	449 ± 365	0.5
Histology	54 (45%)	37 (46%)	17 (43%)	0.85
Urothelial cancer	113 (95%)	73 (92%)	40 (100%)	
Variant histology	6 (5%)	6 (7.6%)	0 (0%)	
T after cystectomy				0.94
≤T2	86 (72%)	58 (73%)	28 (70%)	
≥T3	34 (28%)	22 (28%)	12 (30%)	
Positive lymph nodes	18 (16%)	13 (17%)	5 (13%)	0.82
Clavien–Dindo complications				0.3
1	2 (9.5%)	2 (12%)	0 (0%)	
2	8 (38%)	5 (29%)	3 (75%)	
3	5 (24%)	4 (24%)	1 (25%)	
4	6 (29%)	6 (35%)	0 (0%)	
Hospital stay (days)	21 ± 6.4	21 ± 6.2	22 ± 6.8	0.55
GHS preoperatively	64 ± 23	58 ± 24	76 ± 16	**<0.001**
GHS 3 months	64 ± 20	67 ± 19	57 ± 22	**0.045**
GHS 12 months	68 ± 23	77 ± 16	50 ± 26	**<0.001**

**Table 2:** Incontinence 12 months after surgery of patients with an increase in GHS versus a decrease in GHS. Values presented as mean ± standard deviation and *n* (%). The *t*-test was performed for comparisons between continuous variables and the chi-squared test between categorical variables. The bold cells indicate statistically significant *p*-values. GHS: Global Health Score, ICIQ: International Consultation on Incontinence Questionnaire.

Characteristic	Overall, *n* = 120	Increase in GHS, *n* = 80	Decrease in GHS, *n* = 40	*p*-Value
ICIQ Score	9.0 ± 6.4	7.5 ± 6	12.2 ± 6.1	**<0.001**
Patients with incontinence (≥1 pad/day)	100 (84%)	63 (79%)	37 (95%)	**0.047**

**Table 3:** Baseline characteristics based on the proposed age cutoff of 70 years for ileal orthotopic neobladder. Values presented as mean ± standard deviation or n (%). The *t*-test was performed for comparisons between continuous variables and the chi-squared test between categorical variables. The bold cells indicate statistically significant *p*-values. ASA: American Society of Anesthesiology; BMI: body mass index; GHS: Global Health Score; ICIQ: International Consultation on Incontinence Questionnaire.

Characteristic	Age ≥ 70, n = 46	Age < 70, n = 74	*p*-Value
Male	39 (85%)	58 (78%)	0.53
Age (years)	75.3 ± 3.1	60.5 ± 7.7	**<0.001**
BMI (kg/m^2^)	26.4 ± 4.1	27.0 ± 4.4	0.50
Smokers	32 (71%)	43 (61%)	0.34
Alcohol consumption	22 (50%)	26 (36%)	0.18
Hypertension	25 (54%)	29 (39%)	0.15
Diabetes	15 (33%)	18 (24%)	0.44
Coronary heart disease	13 (28%)	5 (6.8%)	**0.003**
ASA score			**0.018**
1	0 (0%)	4 (5.5%)	
2	10 (22%)	30 (41%)	
3	35 (78%)	39 (53%)	
Operative time (minutes)	209.1 ± 47.5	229.8 ± 54.9	**0.033**
Blood loss (ml)	435.6 ± 447.3	404.8 ± 310.5	0.69
Histology			0.12
Urothelial cancer	46 (100%)	67 (92%)	
Variant histology	0 (0%)	6 (8.2%)	
T after cystectomy			>0.99
≤T2	33 (72%)	53 (72%)	
≥T3	13 (28%)	21 (28%)	
Positive lymph nodes	5 (11%)	13 (18%)	0.39
Clavien–Dindo complications			0.40
1	1 (10%)	1 (9.1%)	
2	3 (30%)	5 (45%)	
3	4 (40%)	1 (9.1%)	
4	2 (20%)	4 (36%)	
Hospital stay (days)	22.4 ± 7.0	20.5 ± 6.0	0.14
ICIQ 12 months	10.4 ± 6.9	8.1 ± 5.9	0.068

**Table 4:** Change from baseline in functioning scores based on the proposed age cutoff of 70 years for ileal orthotopic neobladder. Values presented as mean ± standard deviation. The *t*-test was performed for comparisons between continuous variables.

Functioning Scores	Overall, n = 120	Age ≥ 70, n = 46	Age < 70, n = 74	*p*-Value
Physical functioning	−16.6 ± 29.5	−19.1 ± 31.2	−14.8 ± 28.6	0.6
Role functioning	−15.2 ± 41.1	−25.4 ± 40.8	−7.8 ± 40.4	0.12
Social functioning	−13.0 ± 38.1	−22.7 ± 44.4	−6.3 ± 32.2	0.14
Cognitive functioning	13.0 ± 30.2	18.8 ± 30.7	8.9 ± 29.6	0.23
Emotional functioning	27.0 ± 30.8	26.5 ± 30.9	27.3 ± 31.2	0.92

## Data Availability

The data is not publicly available due to ethical and legal restrictions. However, the data presented in this study are available upon reasonable request from the corresponding author.

## Supplementary Materials

The following supporting information can be downloaded at: https://www.mdpi.com/article/10.3390/jcm13206102/s1, Table S1: Absolute values of the EORTC QLQ-C30 functional and symptom scores preoperatively, 3 and 12 months postoperatively of patients with an increase in the GHS versus a decrease in the GHS. GHS: Global Health Score; Table S2: Absolute values of the EORTC QLQ-C30 functional and symptom scores preoperatively, 3 and 12 months postoperatively based on the proposed age cutoff of 70 years for ileal orthotopic neobladder.

## References

[1] Richters A., Aben K.K.H., Kiemeney L.A.L.M. (2020). The global burden of urinary bladder cancer: An update. World J. Urol..

[2] Pyrgidis N., Volz Y., Ebner B., Kazmierczak P.M., Enzinger B., Hermans J., Buchner A., Stief C., Schulz G.B. (2024). The effect of hospital caseload on perioperative mortality, morbidity and costs in bladder cancer patients undergoing radical cystectomy: Results of the German nationwide inpatient data. World J. Urol..

[3] Klemm J., Fisch M., Laukhtina E., Dahlem R., Shariat S.F., Vetterlein M.W. (2024). Continent diversion is losing its momentum: A nationwide trend analysis from Germany 2005–2021. BJU Int..

[4] Pyrgidis N., Sokolakis I., Haltmair G., Hatzichristodoulou G. (2023). The Short- and Long-Term Effect of Radical Cystectomy in Frail Patients With Bladder Cancer. Clin. Genitourin. Cancer.

[5] Cerruto M.A., D’Elia C., Siracusano S., Gedeshi X., Mariotto A., Iafrate M., Niero M., Lonardi C., Bassi P., Belgrano E. (2016). Systematic review and meta-analysis of non RCT’s on health related quality of life after radical cystectomy using validated questionnaires: Better results with orthotopic neobladder versus ileal conduit. Eur. J. Surg. Oncol..

[6] Volz Y., Eismann L., Pfitzinger P., Westhofen T., Ebner B., Jokisch J.-F., Buchner A., Schulz G., Schlenker B., Karl A. (2022). Long-term Health-related Quality of Life (HRQOL) After Radical Cystectomy and Urinary Diversion—A Propensity Score-matched Analysis. Clin. Genitourin. Cancer.

[7] Pyrgidis N., Sokolakis I., Haltmair G., Hatzichristodoulou G. (2022). The effect of urinary diversion on renal function after cystectomy for bladder cancer: Comparison between ileal conduit, orthotopic ileal neobladder, and heterotopic ileocecal pouch. World J. Urol..

[8] Berger I., Martini T., Wehrberger C., Comploj E., Ponholzer A., Wolfgang M., Breinl E., Dunzinger M., Hofbauer J., Höltl W. (2014). Perioperative complications and 90-day mortality of radical cystectomy in the elderly (75+): A retrospective, multicentre study. Urol. Int..

[9] Witjes J.A., Bruins H.M., Cathomas R., Compérat E.M., Cowan N.C., Gakis G., Hernández V., Espinós E.L., Lorch A., Neuzillet Y. (2021). European Association of Urology Guidelines on Muscle-invasive and Metastatic Bladder Cancer: Summary of the 2020 Guidelines. Eur. Urol..

[10] Cerruto M.A., D’Elia C., Siracusano S., Saleh O., Gacci M., Cacciamani G., De Marco V., Porcaro A.B., Balzarro M., Niero M. (2018). Health-Related Quality of Life after Radical Cystectomy for Bladder Cancer in Elderly Patients with Ileal Orthotopic Neobladder or Ileal Conduit: Results from a Multicentre Cross-Sectional Study Using Validated Questionnaires. Urol. Int..

[11] von Elm E., Altman D.G., Egger M., Pocock S.J., Gotzsche P.C., Vandenbroucke J.P. (2007). Strengthening the Reporting of Observational Studies in Epidemiology (STROBE) statement: Guidelines for reporting observational studies. BMJ.

[12] Aaronson N.K., Ahmedzai S., Bergman B., Bullinger M., Cull A., Duez N.J., Filiberti A., Flechtner H., Fleishman S.B., De Haes J.C.J.M. (1993). The European Organization for Research and Treatment of Cancer QLQ-C30, a quality-of-life instrument for use in international clinical trials in oncology. J. Natl. Cancer Inst..

[13] Shariat S.F., Sfakianos J.P., Droller M.J., Karakiewicz P.I., Meryn S., Bochner B.H. (2010). The effect of age and gender on bladder cancer: A critical review of the literature. BJU Int..

[14] Chang S.S., Bochner B.H., Chou R., Dreicer R., Kamat A.M., Lerner S.P., Lotan Y., Meeks J.J., Michalski J.M., Morgan T.M. (2017). Treatment of Non-Metastatic Muscle-Invasive Bladder Cancer: AUA/ASCO/ASTRO/SUO Guideline. J. Urol..

[15] Imbimbo C., Mirone V., Siracusano S., Niero M., Cerruto M.A., Lonardi C., Artibani W., Bassi P., Iafrate M., Racioppi M. (2015). Quality of Life Assessment With Orthotopic Ileal Neobladder Reconstruction After Radical Cystectomy: Results From a Prospective Italian Multicenter Observational Study. Urology.

[16] Crozier J., Hennessey D., Sengupta S., Bolton D., Lawrentschuk N. (2016). A Systematic Review of Ileal Conduit and Neobladder Outcomes in Primary Bladder Cancer. Urology.

[17] Yang L.S., Shan B.L., Shan L.L., Chin P., Murray S., Ahmadi N., Saxena A. (2016). A systematic review and meta-analysis of quality of life outcomes after radical cystectomy for bladder cancer. Surg. Oncol..

[18] Kretschmer A., Grimm T., Buchner A., Stief C.G., Karl A. (2016). Prognostic features for quality of life after radical cystectomy and orthotopic neobladder. Int. Braz. J. Urol..

[19] Kretschmer A., Grimm T., Buchner A., Jokisch F., Ziegelmüller B., Casuscelli J., Schulz G., Stief C.G., Karl A. (2020). Midterm Health-related Quality of Life After Radical Cystectomy: A Propensity Score-matched Analysis. Eur. Urol. Focus.

[20] Novara G., Ficarra V., Minja A., De Marco V., Artibani W. (2010). Functional results following vescica ileale Padovana (VIP) neobladder: Midterm follow-up analysis with validated questionnaires. Eur. Urol..

[21] Veskimäe E., Neuzillet Y., Rouanne M., MacLennan S., Lam T.B.L., Yuan Y., Compérat E., Cowan N.C., Gakis G., van der Heijden A.G. (2017). Systematic review of the oncological and functional outcomes of pelvic organ-preserving radical cystectomy (RC) compared with standard RC in women who undergo curative surgery and orthotopic neobladder substitution for bladder cancer. BJU Int..

