# Supplementary material for: Radical Cystectomy with Ileal Orthotopic Neobladder after 70 Years Leads to Worse Health-Related Quality of Life
Source: J Clin Med. 2024 Oct 13;13(20):6102. doi: 10.3390/jcm13206102 (PMC11508695; doi:10.3390/jcm13206102)
Supplement: Supplementary file 1 [file jcm-13-06102-s001.zip › jcm-3184218-supplementary.pdf]

Supplementary Table S1 - *Absolute values of the EORTC QLQ-C30 functional and symptom scores preoperatively, 3 and 12 months postoperatively of patients with an increase in the GHS versus a decrease in the GHS. GHS: Global Health Score*

**EORTC QLQ-C30 Scores**	**Overall** n = 120	**Increase of GHS**, n = 80	**Decrease of GHS**, n = 40	**p-value**
**Functional scores preoperatively**				
Physical functioning score	90.6 ± 16.4	89.6 ± 18.2	92.5 ± 11.8	0.29
Role functioning score	81.4 ± 30.0	78.1 ± 32.7	87.9 ± 22.6	0.058
Cognitive functioning score	91.1 ± 17.8	90.2 ± 19.1	92.9 ± 15.0	0.40
Emotional functioning score	67.4 ± 26.5	64.7 ± 27.6	72.9 ± 23.5	0.092
Social functioning score	79.0 ± 27.7	75.2 ± 30.0	86.7 ± 20.7	**0.016**
**Symptom scores preoperatively**				
Pain score	19.2 ± 27.7	21.9 ± 29.6	13.8 ± 22.9	0.10
Fatigue score	20.7 ± 22.8	23.6 ± 25.9	15.0 ± 13.2	**0.017**
Insomnia score	24.3 ± 31.9	26.2 ± 34.0	20.5 ± 27.2	0.33
Appetite loss score	8.4 ± 20.5	10.4 ± 23.5	4.3 ± 11.3	0.056
Nausea/Vomiting score	2.5 ± 9.1	3.5 ± 10.8	0.4 ± 2.6	**0.017**
Constipation score	5.3 ± 14.3	5.4 ± 14.5	5.0 ± 14.2	0.88
Diarrhea score	10.6 ± 23.3	9.6 ± 23.2	12.8 ± 23.7	0.48
**Functional scores 3 months postoperatively**				

**EORTC QLQ-C30 Scores**	**Overall** n = 120	**Increase of GHS**, n = 80	**Decrease of GHS**, n = 40	**p-value**
Physical functioning score	65.5 ± 29.2	67.2 ± 28.1	61.6 ± 31.9	0.54
Role functioning score	49.4 ± 39.5	52.2 ± 40.5	42.7 ± 37.5	0.41
Cognitive functioning score	76.4 ± 28.6	75.9 ± 28.9	77.5 ± 28.8	0.85
Emotional functioning score	62.0 ± 30.4	61.0 ± 32.0	64.2 ± 27.1	0.70
Social functioning score	57.0 ± 33.7	59.2 ± 32.3	52.0 ± 37.2	0.49
**Symptom scores 3 months postoperatively**				
Pain score	30.3 ± 36.2	28.1 ± 35.7	35.3 ± 37.7	0.51
Fatigue score	33.7 ± 24.8	33.1 ± 26.7	34.9 ± 20.9	0.74
Insomnia score	37.0 ± 39.9	36.8 ± 41.6	37.3 ± 37.0	0.97
Appetite loss score	25.9 ± 35.9	28.1 ± 37.6	20.8 ± 31.9	0.48
Nausea/Vomiting score	10.9 ± 22.7	10.1 ± 21.4	12.7 ± 26.0	0.72
Constipation score	23.6 ± 33.7	21.9 ± 31.3	27.5 ± 39.5	0.61
Diarrhea score	23.6 ± 33.1	23.7 ± 34.6	23.5 ± 30.7	0.99
**Functional scores 12 months postoperatively**				
Physical functioning score	73.5 ± 24.4	78.1 ± 22.4	65.3 ± 26.2	0.076
Role functioning score	62.7 ± 34.4	67.1 ± 34.4	55.0 ± 33.8	0.21

**EORTC QLQ-C30 Scores**	**Overall** n = 120	**Increase of GHS**, n = 80	**Decrease of GHS**, n = 40	**p-value**
Cognitive functioning score	79.7 ± 25.2	83.8 ± 21.9	72.5 ± 29.3	0.14
Emotional functioning score	65.6 ± 29.4	66.4 ± 29.0	64.2 ± 30.7	0.79
Social functioning score	66.4 ± 32.6	67.6 ± 31.2	64.2 ± 35.6	0.72
**Symptom scores 12 months postoperatively**				
Pain score	21.9 ± 28.2	20.6 ± 25.3	24.2 ± 33.1	0.68
Fatigue score	41.2 ± 30.9	39.0 ± 30.3	45.0 ± 32.3	0.51
Insomnia score	34.0 ± 33.3	29.2 ± 30.2	41.7 ± 37.3	0.22
Appetite loss score	19.1 ± 28.7	18.6 ± 28.7	20.0 ± 29.4	0.87
Nausea/Vomiting score	9.7 ± 22.4	8.6 ± 22.6	11.7 ± 22.4	0.63
Constipation score	19.1 ± 32.1	15.7 ± 28.7	25.0 ± 37.3	0.34
Diarrhea score	17.3 ± 26.5	14.7 ± 26.2	21.7 ± 27.1	0.36

Supplementary Table S2 - *Absolute values of the EORTC QLQ-C30 functional and symptom scores preoperatively, 3 and 12 months postoperatively based on the proposed age cutoff of 70 years for ileal orthotopic neobladder*

**EORTC QLQ-C30 scores**	**Overall** n = 120	**Age ≥ 70**, n = 46	**Age < 70**, n = 74	**p-value**
**Functional scores preoperatively**				
Physical functioning score	90.6 ± 16.4	91.7 ± 14.6	89.8 ± 17.5	0.52
Role functioning score	81.4 ± 30.0	85.5 ± 24.5	78.8 ± 32.9	0.20
Cognitive functioning score	91.1 ± 17.8	93.5 ± 15.9	89.6 ± 18.9	0.23
Emotional functioning score	67.4 ± 26.5	71.6 ± 27.1	64.9 ± 25.9	0.18
Social functioning score	79.0 ± 27.7	85.9 ± 20.2	74.8 ± 30.9	**0.019**
**Symptom scores preoperatively**				
Pain score	19.2 ± 27.7	13.8 ± 25.4	22.5 ± 28.7	0.084
Fatigue score	20.7 ± 22.8	21.3 ± 24.1	20.4 ± 22.1	0.85
Insomnia score	24.3 ± 31.9	14.8 ± 24.2	30.1 ± 34.8	**0.006**
Appetite loss score	8.4 ± 20.5	4.4 ± 11.5	10.8 ± 24.1	0.055
Nausea/Vomiting score	2.5 ± 9.1	1.1 ± 4.2	3.4 ± 11.0	0.11
Constipation score	5.3 ± 14.3	9.4 ± 19.5	2.7 ± 9.2	**0.032**
Diarrhea score	7.2 ± 16.8	7.2 ± 17.1	7.2 ± 16.8	>0.99
**Functional scores 3 months postoperatively**				
Physical functioning score	65.5 ± 29.2	60.0 ± 32.6	68.1 ± 27.4	0.37

**EORTC QLQ-C30 scores**	**Overall** n = 120	**Age ≥ 70**, n = 46	**Age < 70**, n = 74	**p-value**
Role functioning score	49.4 ± 39.5	46.1 ± 38.0	50.9 ± 40.6	0.67
Cognitive functioning score	76.4 ± 28.6	72.2 ± 29.7	78.4 ± 28.3	0.47
Emotional functioning score	62.0 ± 30.4	56.9 ± 26.4	64.4 ± 32.2	0.37
Social functioning score	57.0 ± 33.7	55.6 ± 33.8	57.7 ± 34.2	0.83
Pain score	30.3 ± 36.2	36.1 ± 38.5	27.5 ± 35.2	0.43
Fatigue score	33.7 ± 24.8	34.3 ± 27.3	33.3 ± 23.4	0.86
Insomnia score	37.0 ± 39.9	31.5 ± 37.0	39.6 ± 41.5	0.47
Appetite loss score	25.9 ± 35.9	16.7 ± 28.6	30.6 ± 38.5	0.14
Nausea/Vomiting score	10.9 ± 22.7	11.1 ± 24.9	10.8 ± 21.9	0.97
Constipation score	23.6 ± 33.7	25.9 ± 38.9	22.5 ± 31.5	0.75
Diarrhea score	23.6 ± 33.1	18.5 ± 28.5	26.1 ± 35.3	0.40
**Functional scores 12 months postoperatively**				
Physical functioning score	73.5 ± 24.4	71.0 ± 25.2	75.2 ± 24.1	0.54
Role functioning score	62.7 ± 34.4	62.3 ± 35.3	63.0 ± 34.3	0.94
Cognitive functioning score	79.7 ± 25.2	76.8 ± 26.5	81.8 ± 24.5	0.48
Emotional functioning score	65.6 ± 29.4	68.9 ± 29.0	63.3 ± 29.8	0.49

**EORTC QLQ-C30 scores**	**Overall** n = 120	**Age ≥ 70**, n = 46	**Age < 70**, n = 74	**p-value**
Social functioning score	66.4 ± 32.6	65.9 ± 36.2	66.7 ± 30.5	0.94
**Symptom scores 12 months postoperatively**				
Pain score	21.9 ± 28.2	26.5 ± 32.8	18.8 ± 24.6	0.35
Fatigue score	41.2 ± 30.9	44.4 ± 30.5	38.9 ± 31.4	0.51
Insomnia score	34.0 ± 33.3	38.1 ± 38.4	31.2 ± 29.7	0.49
Appetite loss score	19.1 ± 28.7	16.7 ± 28.6	20.8 ± 29.0	0.60
Nausea/Vomiting score	9.7 ± 22.4	6.5 ± 14.0	12.0 ± 26.9	0.33
Constipation score	19.1 ± 32.1	25.8 ± 37.0	14.6 ± 28.0	0.24
Diarrhea score	17.3 ± 26.5	16.7 ± 22.4	17.7 ± 29.3	0.88
